# Aids to Nursing

**Published:** 1888-03-10

**Authors:** M. M. Basil

**Affiliations:** Author of "The Commoner Accidents to Life and Limb"


### Aids to Nursing.

By M. M. Basil, M.A., M.B., C.M., Author of "The Commoner Accidents to Life and Limb."

(*Continued from p.* 380.)

### Bed and Bedding.

We have now to consider what may be called *special beds*, that is, beds in which definite modifications are introduced to meet certain requirements.

The *rheumatic bed.* Here the bedstead must be particularly firm—by preference a wooden one—since the least shaking is agony to the patient. Most medical men prefer to have a patient with acute rheumatism placed between blankets; and as the tendency to bed-sores is very slight in such cases, there is no objection to under-blankets. The rheumatic patient will be subjected to repeated examination about the region of the heart; you should therefore get the bed-gown so made that the chest can be fully seen without much disturbance to the patient. If any of the joints are to be repeatedly attended to, it would also be well to slit up the clothing and attach tapes, so that by untying a few slip-knots the parts can be got at without any movement of the limb. There is a general principle worth remembering, viz., that when any part of the body is to be frequently exposed, you should so arrange the clothing that the portion may be rendered accessible without much disturbance. In pneumonia the bed-gown ought to open at the back or side; in diseased limbs the sleeves ought to be slit up and knots used, and so in other cases.

The *tent-bed* is arranged for enabling us to secure an atmosphere nearly saturated with steam. It is used in cases of tracheotomy, some throat diseases, severe bronchitis, etc. Sheets, newspapers, or, preferably, waterproof material, may be used for this purpose. The steam jet must not be directed to the patient's face, but to a point at a safe distance from the head of the bed.

The *fracture bed* is supplied with a pulley or other arrangement for applying extension. If there is no tendency to bedsores it is best to do without a spring-bed. If a spring-bed must be used, then a board may be placed under the mattress at the lower end of the bed. The cradle and the bed-frame are essential parts of a bed in many cases of fractures, peritonitis, chest diseases, ovariotomy, etc.

The *dropsical bed* is arranged to keep the patient more or less in a semi-sitting position. A bed-rest, or a chair with a number of pillows, can be arranged to support the back of the patient uniformly.

The bed for operations for hernia, shortening of the round ligaments, ovariotomy, etc., is so arranged as to relax the abdominal walls as much as possible. The shoulders are well supported, and one or two pillows are placed under the knees to flex the thighs gently. In perineal operations an additional precaution is taken to prevent the soiling of the bedding under the pelvis.

The making of the bed for the lying-in condition is not a part of general nursing, but two annoying mistakes are so often made that a word on the subject may be useful. It must be remembered that in the arrangement of under-sheets, blankets, mackintosh, etc., two distinct objects must be secured: first, to prevent the soiling of the sheets which are to be left under the patient immediately after parturition; secondly, to prevent the soiling of the mattress by discharges during the first few days after the event. These two objects must be kept in view, whatever the modifications that you may make to suit each case, and the directions of the doctor under whom you are in service. The other point is in reference to the position of the towel usually given to the patient to enable her to bring the abdominal muscles into action about the end of the second stage of labour. The towel had best be fixed to the lower end of the bed; if tied to the head of the bed, it must, at this critical period, be taken away from the patient. Unless one or other of these precautions is taken, she may reflexly jerk herself towards the head of the bed at considerable risk to herself.

Draw-sheets are used, first, where bleeding or suppuration is expected, as after injuries and operations. In head or neck injuries the pillow must be protected by draw-sheets or a waterproof. The smell of heated indiarubber is, however, a source of considerable annoyance. Secondly, where the sphincters are paralysed, so that there is incontinence of urine or fæces—in this case they must be changed very frequently. If used in small instalments for a long time, they become magazines of filth, and consequently sources of danger.

Water and air cushions should have proper flannel and linen cases. Air-beds should have the air renewed from time to time. Water-beds must not be filled too suddenly, as some patients are very nervous, and others suffer from "seasickness" in such circumstances. The position of the patient must be carefully watched, as once displaced from the centre, he is gradually floated on to the edge of the bed. The temperature of the water must be regularly watched. Dr. Cullingworth mentions 70 deg. Fahr. as the usual temperature; for most patients this is too high. Of course, you must not prick either water or air cushions or beds. To prevent this and many other accidents, you should use none but safety pins, and never in any case prick pins into pillows or bedding.

The air and water beds hitherto in vogue have suffered under many disadvantages, but the tubular air and water beds of Pocock Brothers, 235, Southwark Bridge, London, appear to be contrived very ingeniously to remove most of the usual shortcomings. Damage to one cylinder will not necessitate change of the whole bed, ventilation is less likely to be interfered with, and irritating discharges will not be long in contact with the skin. In cases of some nervous diseases, especially in the third stage of the general paralysis of the insane, where nursing is so very difficult, they would probably be of signal service. Where the cold pack or cold effusion is to be employed, these beds would enable one to carry the procedure out efficiently and neatly.

Bed-sores must be considered in immediate connexion with the subject in hand, as almost invariably it is from neglecting to observe one or several of the points we have just examined that such ulcers are formed. Some people speak of them as being unavoidable accidents, and describe them as "acute decubitus," "tropho-neuroses," etc. These terms may be serviceable cloaks to hide shortcomings or act as ornaments to conceal ignorance, but must not be used to justify the frequent occurrence of bed-sores. To believe in "acute bedsores" is to believe in what is acutely nonsensical. It is well for you to be thoroughly convinced that, except in very rare diseases, they are invariably preventible accidents. Every case under your care should be looked upon by you as a failure of nursing, in whatever way you may have it justified for the time being. Every case where a bed-sore has occurred is a problem in nursing, whose solution you have failed to discover. Their frequent occurrence in an ordinary ward is undoubtedly a sign of utter incapacity on the part of the nurse in charge. Bed-sores are things that you rarely see, provided you diligently and persistently look for them; on the other hand, you will certainly see them oftener than you wish if you are not on the look-out for them.

I shall say little as to their strictly surgical treatment, since that is not in your province. Prevention here is better than cure in more senses than usual. The trouble necessary in warding them off is infinitesimally small compared to the care which their treatment will require at your hands. Their prevention, therefore, will mainly engage our attention. The chief points to attend to for this object are:

First, a proper and even distribution of pressure. No part of the body must be allowed to be pressed unduly for any length of time. In ordinary conditions we constantly move about more or less, so that the weight of the body is not thrown on any one part except for a short period. This constant movement, however, is impeded or absent in patients who are very weak, comatose, or paralysed. It is therefore necessary to change their positions frequently, as otherwise the weight of the body will kill the part on which the patient is lying just as certainly as plaster of Paris bandage will do if it is applied with undue firmness. Spring-beds, water-beds, air-beds and cushions enable you to distribute pressure very efficiently. If you have to nurse a very heavy patient, or a mere living skeleton whose bones require the least temptation to show themselves, you should have no hesitation in demanding a spring-bed.

Secondly, perfect cleanliness and dryness must be insisted upon systematically. Where bed-sores are to be expected, no under-blankets and mackintosh should be used. If, however, india-rubber must be employed, you should be doubly watchful. Crumbs of bread, seams, wrinkles, and rucks in the sheets act as so many murderous weapons to your patient.

Thirdly, watch the skin, and harden it in time. Alcohol in some form or other—as eau de Cologne, brandy, whisky, rectified spirits, etc.—more or less diluted, is very serviceable here. Wash the part clean, then rub it or mop it with the lotion frequently, and allow the spirit to evaporate from the skin before the patient lies on it or has it covered.

When a sore has formed, you must call the medical man's attention to it at once; but you should not cease from attending to the skin around. There is only one point in the treatment of bed-sores which I shall mention. I have known of two cases where death, if not mainly caused, was at any rate very much accelerated by bed-sores over the sacrum extending into the spinal canal. If a bed-sore is getting rapidly larger in spite of the nursing arrangements already mentioned, there should be no delay in placing the patient on a hammock or canvas stretcher, from which a portion has been cut out large enough to allow the ulcer and some healthy skin round it to be freed from all pressure.

*(To be continued.)*

